# Mesenchymal stem cells repress Th17 molecular program through the PD-1 pathway

**DOI:** 10.1186/1479-5876-10-S3-P16

**Published:** 2012-11-28

**Authors:** Patricia Luz-Crawford, Daniele Noël, Fernando Figueroa, Flavio Carrión, Christian Jorgensen, Farida Djouad

**Affiliations:** 1 Inserm, U 844, Montpellier, France; 2 Laboratorio de Immunologia Celular y Molecular, Universidad de Los Andes, Santiago, Chile

## 

MSC display potent suppressive properties initially described a decade ago. Since then, MSC suppressive activities on T-cell effector pathways have been highly investigated. MSC modulate CD4 cell differentiation through different mechanisms depending on culture conditions and display disparate activities on T cells according to their differentiation status. A significant amount of evidence for MSC effects on Th17 cells revealed that MSC could be suppressive under diverse circumstances but also enhance Th17 cell activity under other conditions. In the present study, we investigated the suppressive effects of MSC on Th1 and Th17 subsets of T cells using T cells undergoing Th1 and Th17 polarization or, on mature Th1 and Th17 cells. We showed that MSC inhibited the proliferation of mature Th1 cells and T cells during their differentiation toward Th1 cells. This suppressive effect was maintained in a transwell culture insert, where MSC were plated in the insert, demonstrating the major role played by soluble factors. Using the *transwell* barrier, we observed that MSC decrease the number of T cells undergoing Th17 differentiation whereas they did not affect IL-17 production by mature Th17, demonstrating the need for cell contact for suppressing Th17 cell function. We observed a high level of PD-L1 expression on MSC co-cultured with differentiating or polarized Th1 and Th17 cells. Importantly, using neutralizing antibodies specific for PD-L1 and PD-1, we showed that the mechanisms by which *MSC mediate* Th17 cell repolarization depend on PD-L1 expression on MSC. Taken together, our results demonstrated a cell-to-cell contact depend mechanism in the selective immunosuppression of MSC on mature Th17 cells through up-regulation of PD-L1.

